# Nutrihealth Study: Seasonal Variation in Vitamin D Status Among the Slovenian Adult and Elderly Population

**DOI:** 10.3390/nu12061838

**Published:** 2020-06-19

**Authors:** Maša Hribar, Hristo Hristov, Matej Gregorič, Urška Blaznik, Katja Zaletel, Adrijana Oblak, Joško Osredkar, Anita Kušar, Katja Žmitek, Irena Rogelj, Igor Pravst

**Affiliations:** 1 Nutrition Institute, Tržaška cesta 40, SI-1000 Ljubljana, Slovenia; masa.hribar@nutris.org (M.H.); hristo.hristov@nutris.org (H.H.); anita.kusar@nutris.org (A.K.); katja.zmitek@vist.si (K.Ž.); 2 Biotechnical Faculty, University of Ljubljana, Jamnikarjeva 101, SI-1000 Ljubljana, Slovenia; irena.rogelj@bf.uni-lj.si; 3 National Institute of Public Health, Trubarjeva 2, SI-1000 Ljubljana, Slovenia; matej.gregoric@nijz.si (M.G.); urska.blaznik@nijz.si (U.B.); 4 University Medical Centre Ljubljana, Zaloška cesta 7, SI-1000 Ljubljana, Slovenia; katja.zaletel@kclj.si (K.Z.); adrijana.oblak@kclj.si (A.O.); josko.osredkar@kclj.si (J.O.); 5 Faculty of pharmacy, University of Ljubljana, Aškerčeva cesta 7, SI-1000 Ljubljana, Slovenia; 6 VIST–Higher School of Applied Sciences, Gerbičeva cesta 51A, SI-1000 Ljubljana, Slovenia

**Keywords:** 25(OH)vitamin D, biomarkers, dietary survey, public health, EU Menu, Slovenia, Europe

## Abstract

Several studies conducted around the world showed substantial vitamin D insufficiency and deficiency among different population groups. Sources of vitamin D in the human body include ultraviolet B (UVB)-light-induced biosynthesis and dietary intake, but people’s diets are often poor in vitamin D. Furthermore, in many regions, sun exposure and the intensity of UVB irradiation during wintertime are not sufficient for vitamin D biosynthesis. In Slovenia, epidemiological data about vitamin D status in the population were investigated through a national Nutrihealth study—an extension to the national dietary survey SI.Menu (2017/18). The study was conducted on a representative sample of 125 adult (18–64 years) and 155 elderly (65–74 years old) subjects, enrolled in the study in different seasons. Their vitamin D status was determined by measuring the serum 25-hydroxy-vitamin D (25(OH)D) concentration. Thresholds for vitamin D deficiency and insufficiency were 25(OH)D levels below 30 and 50 nmol/L, respectively. Altogether, 24.9% of the adults and 23.5% of the elderly were found to be vitamin D deficient, while an insufficient status was found in 58.2% and 62.9%, respectively. A particularly concerning situation was observed during extended wintertime (November–April); vitamin D deficiency was found in 40.8% and 34.6%, and insufficient serum 25(OH)D levels were observed in 81.6% and 78.8%, respectively. The results of the study showed high seasonal variation in serum 25(OH)D levels in both the adult and elderly population, with deficiency being especially pronounced during wintertime. The prevalence of this deficiency in Slovenia is among the highest in Europe and poses a possible public health risk that needs to be addressed with appropriate recommendations and/or policy interventions.

## 1. Introduction

Research related to the epidemiology of vitamin D status in different populations is linked to public health concerns due to the high prevalence rates of vitamin D insufficiency and deficiency [1,2]. Several studies conducted in Europe and around the world have shown substantial vitamin D insufficiency and deficiency among different population groups [1,2,3,4,5,6,7,8]. Due to the many roles of vitamin D in human physiology, its low serum concentrations can pose a health risk [3,9]. Vitamin D is a fat-soluble vitamin that is involved in calcium and phosphorus homeostasis, and it therefore plays a crucial role in bone health [10]. While several epidemiological studies have also linked vitamin D deficiency with non-skeletal health outcomes, disease occurrence, immune system function, and reduced life expectancy, the cause–effect evidence from randomized controlled studies remains limited [11,12]. Vitamin D sufficiency varies among and between populations. The elderly are considered an especially vulnerable population group, with a higher prevalence of low vitamin D status and associated health risks [13].

The main sources of vitamin D are skin exposure to ultraviolet B light (UVB) radiation and dietary intake. The amount of vitamin D production via UVB radiation in the skin depends on latitude, season, and time of the day [14,15,16]. It was suggested that sun exposure covers most vitamin D requirements, but in many European countries, sun exposure during most of the wintertime does not lead to the production of vitamin D in the skin, since the intensity of UVB radiation in the sunlight is too low for the efficient cutaneous biosynthesis of cholecalciferol [17,18,19]. Examples of other factors influencing the cutaneous biosynthesis of vitamin D are skin pigmentation, age (especially an age >65 years), and the topical application of sunscreen [14]. On the other hand, the dietary intake of vitamin D with food is commonly low [1,5]. Very few foods are a rich source of vitamin D, and such foods are seldom consumed. The majority of vitamin D intake is, therefore, achieved with foods that are poorer in vitamin D but consumed more regularly [1,20]. Therefore, fortified foods and food supplements also represent an important dietary source of vitamin D [1]. Recommendations for vitamin D intake in the adult population vary widely across Europe [21]. The WHO sets the recommended vitamin D intake at 10 µg/day (400 International Units (IUs); 51–65 years) and 15 µg/day (600 IU; > 65 years) [22], and the daily recommended levels by the European Food Safety Authority (EFSA) and D-A-CH (The nutrition societies of Germany, Austria, and Switzerland) are 15 (600 IU) and 20 µg/day (800 IU) (in the absence of endogenous synthesis), respectively [23,24]. The latter recommendations include the total vitamin D supplied from food and cutaneous biosynthesis. It is estimated that the daily dietary intake of vitamin D in many European populations is well below 10 µg [25,26]. However, because vitamin D status in individuals is particularly affected by the efficiency of cutaneous biosynthesis, dietary intake is not considered a reliable predictor. Therefore, vitamin D status is mainly determined using serum 25-OH vitamin D (25(OH)D) levels [27]. Vitamin D deficiency is usually defined as serum levels below 30 nmol/L (10–12 ng/mL) [24,28,29,30], while serum levels below 50 nmol/L (20 ng/mL) are considered insufficient [24,28,30,31]. On the other hand, a serum 25(OH)D concentration above 75 nmol/L (30 ng/mL) is recommended by the Endocrine Society’s clinical practice guidelines [29].

Slovenia is a country in Central Europe with a latitude between 45° and 46° north, with a population of approximately 2 million people. To date, only two studies have systematically investigated vitamin D deficiency in Slovenia; both were conducted among pregnant women and reported high prevalence rates of vitamin D deficiency [32,33]. Considering the results of the studies conducted in other countries in our region [1,2,5,6,8,30,34], there is a clear need for epidemiological data on the vitamin D status among the Slovenian general population.

Considering these facts, a nutritional Nutrihealth study was conducted as part of the larger research project, “Children’s and adults’ nutrition as a protective or health-risk factor”, which was funded by the Slovenian Research Agency and the Ministry of Health. This Nutrihealth study is an extension of the national dietary SI.Menu survey with a collection of blood and urine samples, thus enabling the assessment of micronutrient status and helping policymakers engage in evidence-based policy decisions. The objective of the present study was to elucidate the seasonal variation in vitamin D status among the Slovenian adult and elderly population.

## 2. Experimental Section

### 2.1. Study Design and Data Collection

The Nutrihealth study was conducted as an upgrade to the Slovenian national dietary survey SI.Menu 2017/2018, which was carried out following the EFSA Guidance on EU Menu Methodology [35]. The complete methodology of the SI.Menu study is detailed elsewhere [36]. In short, the SI.Menu study included nationally representative (age/sex/region) samples of adolescents (10–17 years of age), adults (18–64 years), and the elderly (65–74 years). The recruitment period ran from March 2017 to February 2018, divided into quarters (Q): Q1 (March–May 2017), Q2 (June–August 2017), Q3 (September–November 2017), and Q4 (December 2017–February 2018). The survey contained a food propensity questionnaire (FPQ); two non-consecutive 24 h dietary recalls; information concerning eating habits; consumer habits; food allergies; food supplement use; lifestyle; socio-demographic and socio-economic status; body height; weight; self-evaluated health status; and percentage of fat, water, and fat-free mass measured with a bioimpedance scale. Physical activity levels were assessed using the International Physical Activity Questionnaire (IPAQ) and scored as described by Craig et al. [37]. A subsample of adult participants that completed the SI.Menu study (all participating adults (18–74 years) in Q2, Q3, and Q4) was invited to participate in the Nutrihealth study.

The Nutrihealth study protocol was approved by the Slovenian National Medical Ethics Committee (Ministry of Health, Republic of Slovenia), identification number KME 72/07/16 (approval letter ID 0120-337/2016-4, date of approval: 7 July 2017) and was registered at ClinicalTrials.gov (ID: NCT03284840). The study was performed in compliance with the requirements of the local authorities. All subjects signed a written informed consent form (ICF) before participation in the study. The Nutrihealth study included the collection of fasting blood samples, spot urine samples, and thyroid inspection/palpations and ultrasounds. The collection of biological samples was carried out in local healthcare centers from June 2017 to September 2018. Blood samples were collected on daily basis during regular working hours of the local healthcare centers and transported to a central laboratory at the University Medical Center in Ljubljana, where they were stored at −80 °C until analysis. Determination of the serum 25(OH)D was conducted at the Department of Nuclear Medicine on a complete set of samples after sample collection was completed.

### 2.2. Study Population

While the SI.Menu study was conducted on a nationally representative sample of adolescents, adults, and the elderly; only the adults and elderly were included in the Nutrihealth study. Altogether, 1319 participants (a 62.2% response rate) fully completed the SI.Menu study (484 adolescents, 385 adults, and 450 elderly), and 620 participants (282 adults and 338 elderly) were invited to the Nutrihealth study. Altogether, 394 subjects (183 adults and 211 elderly) signed the consent, and 280 (125 adults and 155 elderly; 68.3% and 73.5%, respectively) provided biological samples. Descriptive characteristics of the final populations are provided in Table 1.

### 2.3. Serum 25(OH)D Concentration

Serum 25(OH)D concentration was measured in human serum at the Department of Nuclear Medicine (University Medical Center, Ljubljana) with the chemiluminescence immunoassay vitamin D total (25-hydroxy-vitamin D) determined on an IDS-iSYS analyzer (Immunodiagnostic Systems, Boldon, UK). The correlation coefficient using the ID-LC-MS/MS method within the assay measuring interval (22.5–246.5 nmol/L) was r = 0.925. Vitamin D status was assigned with consideration of serum 25(OH)D concentration according to the literature: Deficient below 30 nmol/L (12 ng/mL) and insufficient bellow 50 nmol/L (20 ng/mL) [28].

### 2.4. Statistical Analysis

The statistical analysis was conducted using STATA version 13 (StataCorp, Coledge Station, TX, USA). Descriptive characteristics (means, median, and proportions), as well as the proportions of participants with different levels of 25(OH)D, are presented for all participants and per age group. Considering the sampling approach, most of the analyses were done separately for the adults and the elderly. For serum 25(OH)D levels and for the prevalence low serum 25(OH)D (levels below 30/50/75 nmol/L) we used population weighting (sex and age) separately for the sample groups of adults and elderly. As Slovenia lies at latitudes lacking sufficient sunlight to produce cutaneous vitamin D during the extended winter [19], two seasonal periods were used for the statistical analyses: May–October (extended summer) and November–April (extended winter). On the other hand, weighting of the serum 25(OH)D levels in bimonthly periods (January–February, March–April, May–June; July–August; September–October; November–December) was performed on the merged samples of the adults and elderly (*N* = 280). Weighting factors were computed using the iterative proportional fitting method with the Slovenian national census data for July 2017.

The unadjusted mean values of serum 25(OH)D were determined by sex, education, income, season, body mass index (BMI), smoking status, physical activity, and vitamin D supplement use. BMI was defined as lean or normal weight (BMI < 25) and overweight/obese (BMI ≥ 25) and was calculated as weight (kg)/height(m^2^). Data on household monthly net income were used to assign the income status (≤900 €; between 900 € and 1800 €/month; and >1800 €/month). A linear logistic regression analysis was used to investigate the differences between the different sub-populations of both samples. The mean 25(OH)D levels were further adjusted for the above-mentioned variables as possible confounders. The prevalence of adults and elderly with serum 25(OH)D levels less than 30 nmol/L and 50 nmol/L was determined by sex, education, income, season, BMI, smoking status, physical activity, and vitamin D supplement use. A multivariable logistic regression analysis was undertaken with all of the above-mentioned variables per age group to determine the independent predictors of vitamin D insufficiency (serum 25(OH)D level < 50 nmol/L). In all comparisons, significance was considered at *p* < 0.05.

The aggregated data for adults and elderly participants within the extended wintertime (*n* = 164) were used to conduct a prevalence analysis (serum 25(OH)D level < 50 nmol/L) considering the consumption of the selected dietary sources of the vitamin. We used the food propensity questionnaire (FPQ) data (FPQ options: at least once per day, 4–6 times per week, 2–4 times per week, once per week, 1–3 times per month or less, or never) for seafood; saltwater fish; yogurts, sour milk, and curd; and milk to differentiate between regular consumers (a consumption frequency of at least once per week) and non-consumers (a consumption frequency of never).

## 3. Results

As a part of the Nutrihealth study, blood samples were collected from the 280 subjects. Considering the sampling approach, the data were analyzed separately for adults (18–64 years) and the elderly (65–74 years). The study population thus consisted of 125 adults and 155 elderly; the characteristics of both sample groups are summarized in Table 1.

**Figure 1:**
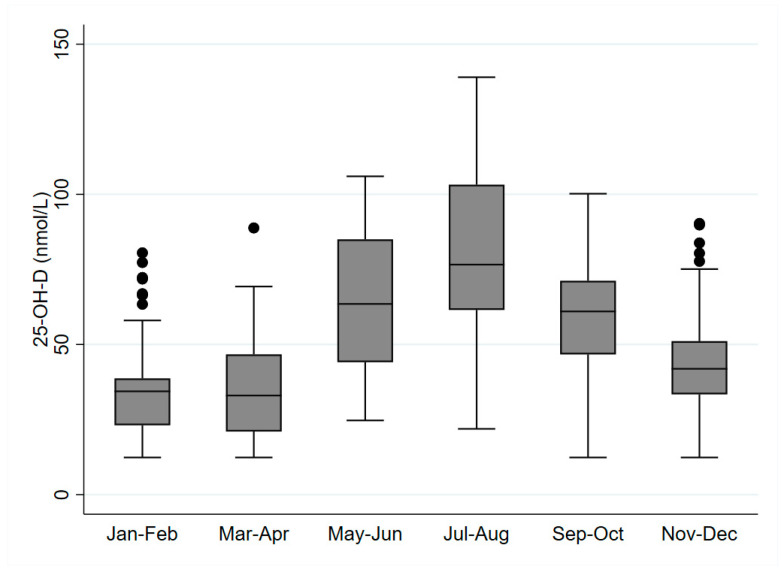
Box plots of the weighted (age and sex) mean serum 25(OH)D concentrations for different bi-monthly periods (*N* = 280) with presentation of outliers (∙).

The yearly population-weighted (age and sex) serum 25(OH)D levels and prevalence of serum 25(OH)D levels below 30/50/75 nmol/L for both populations are presented in Table 2. Critically low serum 25(OH)D levels (<30 nmol/L) were observed in about a quarter of both samples, while about 60% had levels below the recommended 50 nmol/L. Only around a fifth of the population had serum 25(OH)D levels above 75 nmol/L. Notably, we observed seasonal differences in the serum 25(OH)D levels in different periods of the year. Figure 1 presents the population-weighted (age, sex) mean serum 25(OH)D concentrations in two-month intervals, considering the measurements in both samples (*N* = 280). It can be observed that the serum 25(OH)D levels are notably higher between May and October (extended summer) compared to the period between November and April (extended winter).

To provide further insights into the vitamin D status of the population, we calculated seasonal population-weighted serum 25(OH)D levels and prevalence of serum 25(OH)D levels below 30/50/75 nmol/L separately for the extended summer and winter periods (Table 3).

Relatively low levels of vitamin D deficiency (<30 nmol/L) were observed during the extended summer. In adults, the prevalence of deficiency was 2.6% (95%CI: 0.6, 10.2), without notable differences between males and females. On the other hand, 16.1% (95%CI: 4.9, 41.5) of males and 34.6% (19.5–53.6) of females had serum 25(OH)D levels below 50 nmol/L. Among the elderly, the prevalence of vitamin D deficiency during extended summer was somewhat higher; 7.8% of males (95%CI: 3.2, 17.5) and 40.2% of females (95%CI: 28.8, 52.7) had serum 25(OH)D levels below 50 nmol/L. The prevalence of insufficient serum 25(OH)D levels was 27.6% (95% CI: 14.3, 46.5) in males and 51.4% (95%CI: 35.1–67.5) in females.

A notably higher prevalence of vitamin D deficiency was observed during extended winter, with 40.8% (95%CI; 29.0,53.7) of adults and 34.6% (95%CI: 25.4, 45.1) of the elderly having serum 25(OH)D levels below 30 nmol/L. Prevalence of vitamin D deficiency in females and males was 44.5% (95%CI: 29.7, 60.3) vs. 37.1% (95%CI: 20.4, 57.6) for adults and 40.9% (95%CI: 27.4, 56.0) vs. 27.7% (95%CI: 16.7, 42.2) for the elderly, respectively. During the extended winter period, about four-fifths of both populations did not meet the recommended serum 25(OH)D level of 50 nmol/L.

The reported results clearly show that serum 25(OH)D levels are affected by the season. A multivariable logistic regression analysis was done using the season, sex, residential area, education, family net income, BMI, smoking status, physical activity, and use of vitamin D supplements as possible independent predictors of the prevalence of insufficient vitamin D status. Table 4 presents a sample of serum 25(OH)D levels below 50 nmol/L and the adjusted odds ratios (ORs) for both study populations. In line with previous observations, season was identified as a significant predictor of vitamin status in both the adult and elderly populations (*p* < 0.0001). The odds ratios for insufficient vitamin D status were 34.9 for adults (95%CI: 9.2, 132.5) and 10.3 for the elderly (95%CI: 4.1, 26.1), respectively. For the elderly, only body mass index was identified as an additional significant predictor (*p* = 0.014), with subjects possessing a lower BMI having a lower odds ratio for insufficient vitamin D status (OR 0.3; 95%CI: 0.1, 0.8). On the other hand, sex *(p* = 0.041) and physical activity (0.048) were identified as significant parameters in the adult population, with a higher OR for females (OR 3.4; 95%CI: 1.1–10.7) and for those with a low level of physical activity (OR 5.6; 95%CI: 9.22–22.7; *p* = 0.016). Similar results were observed in the regression analysis of the sample mean serum 25(OH)D levels (Supplementary Materials Table S1). After adjustment for the above mentioned confounding factors, the mean serum 25(OH)D concentrations were 49.9 (95% CI: 45.3, 54.6) and 47.7 nmol/L (95% CI: 43.9, 51.4) for adults and the elderly, respectively, with higher levels in extended summer than in extended winter. It should be noted that season was again a significant parameter in both populations (*p* < 0.001), along with BMI (*p* = 0.0065) among the elderly population and sex (*p* = 0.031) among the adult population. Additionally, for adults, smoking status (*p* = 0.011) was also identified as a significant parameter, while physical activity was not significant.

**Figure 2:**
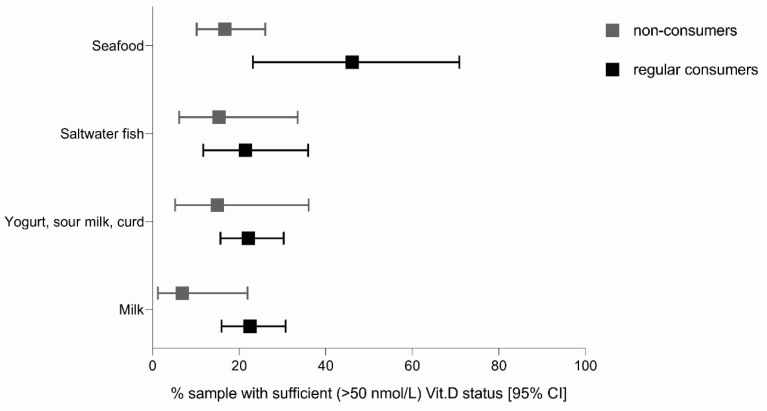
Proportion (%) of subjects with sufficient vitamin D status (serum 25(OH)D level >50 nmol/L) for (non)consumers of selected dietary sources of vitamin D (aggregated data for adults and the elderly during the extended winter period (November–April); *n* = 164).

To gain insight into the importance of dietary habits on vitamin D status, we next studied vitamin D status in relation to the consumption frequency of important dietary sources of vitamin D. To avoid the cofounding of sun-exposure-related vitamin biosynthesis, this analysis was done with the exclusion of extended summertime measurements. We used aggregate data for the adult and elderly populations (*n* = 164); food consumption frequency was measured using a survey-based food propensity questionnaire. Figure 2 presents the proportion of study subjects with serum 25(OH)D concentrations above 50 nmol/L for non-consumers and regular consumers of selected dietary sources of vitamin D. While the differences did not reach statistical significance, in all selected food categories, the proportions of vitamin D sufficiency were higher for regular consumers than for non-consumers. For example, 46.2% (95%CI: 23.2, 70.9) of regular consumers of seafood had a serum 25(OH)D level above 50 nmol/L, while for non-consumers, this was the case in only 15.4% (95%CI: 6.1–33.5).

## 4. Discussion

Epidemiological studies on the vitamin D status in various populations are receiving increased attention, mostly due to the various associations of vitamin D deficiency with health outcomes [1,2]. This is also the case in Europe [1,2,3,4,5,6,7,8]. In a recent ODIN study carried out among European residents living across a latitude gradient of 35° N to 69° N, about 13% of people were vitamin D deficient, and 40% were insufficient [5]. Previous research suggests that mid-latitude countries can have an even higher prevalence of deficiency than northern countries, such as Norway, Iceland, and Finland [2].

Slovenia is also a mid-latitude country (latitude 45° and 46° North) but with very limited epidemiological data on vitamin D status. Studies conducted among pregnant women showed high rates of vitamin D deficiency [32,33], indicating the need for a nationally representative study. This study was designed as an extension to the cross-sectional national dietary survey SI.Menu 2017/2018 [30].

It should be noted that the optimal serum 25(OH)D concentration remains subject to different opinions. According to the Institute of Medicine (IOM) [38], EFSA [24], and European Calcified Tissue Society [30], the recommended serum level of 25(OH)D should be above 50 nmol/L for skeletal health benefits, while the Endocrine Society [29] recommends concentrations above 75 nmol/L. In this paper, we use the term “vitamin D deficiency” for serum 25(OH)D levels below 30 nmol/L and “vitamin D insufficiency” for levels below 50 nmol/L. The reported prevalence for vitamin D deficiency in this study was 24.9% and 23.5% among the adults (18–64 years old) and elderly (65–74 years old), respectively. Insufficiency was observed in 58.2% of adults and 62.9% of the elderly population, which ranks Slovenia among the countries with the highest vitamin D insufficiency in Europe [2,5,8,30]. The prevalence of insufficiency was high even when comparing countries with similar latitudes. Germany (47–55° N; 18–79 years) reported 54.5% prevalence [2] and France (43-49° N; 18–89 years) 34.6% [39], while a >82% prevalence was reported for the Ukraine (44–52° N; 20–95 years) [40]. The vitamin D insufficiency prevalence reported here is also high when compared to some other countries across the world. A US study revealed a prevalence of 41.7% (36.0–47.6) and 41.1% (37.4–44.6) for adults (<65 years old) and the elderly (≥65 years old), respectively [41], while in China, the prevalence was 55.9% in adults [42] and 34.3% in the elderly population (>60 years old) [43]. A somewhat lower prevalence (22.7%, 95%CI: 20.5, 35.1) was observed in the Australian adult and elderly population [44]. According to the WHO, vitamin D insufficiency can be classified as a severe health problem since it affects more than 40% of the global population [45], and Slovenia, with many other countries, falls into this category.

The main source of vitamin D is dermal synthesis induced by UVB [46,47]. UVB availability varies highly during the year, and the amplitude of variation is mostly dependent on latitude [19]. Therefore, this study was also focused on investigating the seasonal variation of vitamin D status in the adult and elderly populations. We split the observation time into two periods: extended winter (November–April) and extended summer (May–October) since the winter and springtime vitamin D statuses decline and reach their nadir typically in late winter or early spring [48]. This is demonstrated in Figure 1, which shows the peak values of 25(OH)D concentrations in July and August. These values then steadily decline until late winter, with the difference of the mean values between extended winter and extended summer at almost 30 nmol/L.

The adjusted mean 25(OH)D levels were significantly lower in the extended winter period, and the odds ratios for insufficient vitamin D status were significantly lower in extended summer, both in adults and the elderly. The population weighted prevalence of vitamin D deficiency during extended winter in adults was 40.8% (95% CI: 29.0–53.7), which was more than 10 times higher than that during extended summer, when prevalence of deficiency was only 2.6% (95% CI: 0.6–10.2). At the same time, the prevalence of vitamin D insufficiency rose from 25.3% (95% CI: 14.8, 39.9) in the extended summer to 81.6% (95% CI:69.4, 89.7) in the extended winter. This means that four out of five people will be vitamin D deficient during the winter period. Such seasonal variations are common in countries at this latitude [19,43,44,47,49,50,51,52].

The literature data show that the elderly are more susceptible to lower 25(OH)D concentrations [5,13,30,34,49,53,54], likely due to their decreased cutaneous vitamin D production, sun-avoidance behaviors [55] (and consequently reduced sun exposure [56]), and lower dietary intake of vitamin D [57]. Sampling in our study was, therefore, done with a stronger focus on the elderly population. Interestingly, the prevalence of vitamin D deficiency in the summer period was notably higher than in adults (7.8% vs. 2.6%), but this was not the case during the extended wintertime. However, the elderly population in the SI.Menu study was represented by free-living individuals, excluding those living in elderly institutions. We suspect that the lower seasonal variability in vitamin D status in the elderly population is related to their sun-avoidance behavior. These findings support previous results and highlight that season is a major factor for determining vitamin D status.

The mean serum 25(OH)D levels in this study for the adult population were 50.7 nmol/L (95% CI: 45.4, 56.0) and 47.7 nmol/L (95% CI: 43.9, 51.5) for the elderly, which are lower values in comparison to most countries in the neighborhood. The reference mean values for the adult and elderly participants were 50.1 nmol/L in Germany [2], 52.3 nmol/L in Austria [34], and 60 nmol/L in France [39], while Croatian postmenopausal women had a mean value of 64.9 nmol/L [58]. In adults (but not in the elderly), we observed significant differences in the mean vitamin D values between sexes, with males having higher mean values than females at 55.3 (95% CI: 46.4, 64.1) and 46.2 (95%CI: 40.4, 51.9) nmol/L, respectively. These findings are consistent with those of some other studies [34,44,47,49,59], but there are also studies with opposite observations (India, Saudi Arabia) [60,61].

Adiposity is also a well-known risk factor for vitamin D deficiency/insufficiency [4]. The bioavailability of vitamin D from cutaneous and dietary sources decreases through its deposition in body fat compartments [62,63] and can also be lower due to reduced outdoor physical activity [50,64]. In our sample, 60.8% of adults and 70.3% of the elderly population were overweight or obese, which may have affected the high prevalence of vitamin D deficiency/insufficiency. In the elderly population, we observed a particularly high (and statistically significant; *p* = 0.014) odds ratio (OR 10.3; 95%CI: 4.10, 26.7) for vitamin D insufficiency in overweight/obese subjects in comparison to those with normal or lean BMIs. Considering our inability to observe this trend in the adult population, we should note that the sample of elderly participants (65–74 years) was much more homogenous in their age interval than the adult subjects, whose age interval was much higher (18–65 years). On the other hand, smoking was identified as a significant predictor of mean serum 25(OH)D levels, with current smokers having a lower means compared to non- or ex-smokers, which is in agreement with other studies [44,47]. Interestingly, in our study, the use of vitamin D supplements was not identified as a significant predictor of vitamin D status. While we observed higher mean serum 25(OH)D levels and a lower prevalence of vitamin D insufficiency in the group of supplement users, the proportion of vitamin D supplement users was very low (9% in adults and 8% in the elderly), and the doses of vitamin D are commonly too low to achieve sufficient vitamin D intake. For example, even within supplement (vitamin D) users, the vitamin D level in the majority of the obtained serum samples was below the recommended level of 50 nmol/L (59.7% of adults and 64.1% of the elderly).

During an extended winter period with low or no UVB-induced vitamin D biosynthesis, vitamin D becomes essential, with diet providing its only source [5,19]. In subjects (adult and elderly) whose blood samples were collected during the extended winter period, food propensity questionnaire data were used to identify some dietary habits, thus affecting vitamin D status (Figure 1). Regular consumers of seafood had a notably higher prevalence of vitamin D sufficiency compared to non-consumers of seafood, while the difference for the other food groups was less obvious.

The strengths of this study include the recruitment of a national population-based sample of Slovenian adults (18 to 64 years) and elderly (65 to 74 years) participants with a measurement of serum 25(OH)D levels, which was done centrally in one laboratory using the same methodology. However, some limitations of this study should be also noted. First, while the sample was nationally representative, the sample size limited our ability to identify additional parameters affecting vitamin D status, particularly those related to dietary habits. Secondly, this is a cross-sectional study. Thus, the causality between vitamin D deficiency and its determinants could not be determined. Third, we did not obtain data on the prevailing weather conditions, ozone, or sun exposure practices, including clothing type in summer, time spent outdoors, and sunscreen use. Therefore, specific analyses were done on the samples obtained during extended wintertime to avoid the effects of UV-B induced vitamin D biosynthesis. It should be noted, however, that even if data on sun exposure were available, any compensation for vitamin D biosynthesis would be very difficult. Our study results also showed that season is the strongest parameter affecting vitamin D status, wich seriously limits the ability to identify other predictors of vitamin D status during the extended summer, where biosynthesis presents the most important source of vitamin D. Another study limitation is that the national dietary survey SI.Menu was not conducted in a way that facilitated the reliable estimation of dietary vitamin D intake at the individual level. Therefore, the food propensity questionnaire data were used only to provide some insights about the vitamin D supply with consideration of the (non)consumption of specific food types, while the effects of total daily dietary vitamin D intake were not investigated.

## 5. Conclusions

Ultimately, we observed considerable seasonal variability in the vitamin D status of both the adult and elderly populations. The highest vitamin D deficiency rates were observed during extended wintertime (November–April; 40.8% of adults and 34.6% of the elderly). During that time, about 80% of the population had insufficient vitamin D levels (<50 nmol/L). In addition to season, other important factors affecting vitamin D status included sex and physical activity for adults and BMI for the elderly population. The high prevalence of vitamin D deficiency and insufficiency in Slovenia during extended wintertime poses a possible public health risk, which should be addressed with appropriate recommendations and/or policy interventions. This could be done at the national or European Union level. Considering that we observed relatively low serum 25(OH)D levels also on those reporting use of vitamin D supplements, further studies should focus into identification of existing supplementation practices, to identify usual dosages and forms of vitamin D that are being used by different populations.

## Figures and Tables

**Table 1:** Descriptive characteristics of the population.

Variable		Adults	Elderly
		(18–64 Years Old)	(65–74 Years Old)
		*N* = 125	*N* = 155
Age (mean ± SD)		46.5 (13.2)	68.6 (2.8)
Residential area (%)	village	50.4	54.2
	town	15.2	15.5
	city	34.4	30.3
Sex (%)	male	41.6	49
	female	58.4	51
Education (%)	primary school	8.8	19.4
	high school	60	55.5
	higher education	31.2	25.1
Monthly net income (%)	≤900 €	20.3	32.2
	900–1800 €	47.8	55
	>1800 €	31.9	12.8
Season (%)	November–April	58.4	58.7
	May–October	41.6	41.3
BMI (mean ± SD)		27.6 (5.5)	27.9 (4.7)
BMI (%)	<25	39.2	29.7
	≥25	60.8	70.3
Smoking status (%)	current smoker	17.6	11.6
	ex-/non-smoker	82.4	88.4
Physical activity * (%)	low level	31.2	33.1
	moderate level	32	31.8
	high level	36.8	35.1
Vitamin D supplement use (%)	users	8.8	8.4
	non-users	91.2	91.6

Notes: SD = standard deviation; BMI = body mass index; * physical activity according to International Physical Activity Questionnaire (IPAQ).

**Table 2:** Yearly population-weighted (age, sex) serum 25(OH)D levels and prevalence of serum 25(OH)D levels <30, <50, and <75 nmol/L (95% CI) for adults (18–64 years) and elderly (65–74 years).

	*N* (%) *	Serum 25(OH)D Level (nmol/L)			Prevalence (%)		
		Mean	S.E.	Median	<30 nmol/L	<50 nmol/L	<75 nmol/L
Adults	125 (100)	50.7 (45.4–56.0)	2.7	45.3	24.9 (17.5–34.1)	58.2 (48.5–67.3)	83.3(74.9–89.2)
-Male	52 (41.6)	55.3 (46.4–64.1)	4.5	50.7	22.8 (12.6–37.8)	50.0 (35.4–64.6)	79.6 (65.7–88.8)
-Female	73 (58.4)	46.2 (40.4–51.9)	2.9	43.8	27.0 (17.6–39.0)	66.4 (54.3–76.7)	86.9 (76.1–93.2)
Elderly	155 (100)	47.7 (43.9–51.5)	1.9	42.4	23.5 (17.4–30.9)	62.9 (54.9–70.2)	84.4 (77.8–89.4)
-Male	76 (49.0)	48.2 (43.0–53.3)	2.6	42.1	19.1 (11.8–29.5)	60.3 (48.7–70.7)	84.9 (74.7–91.5)
-Female	79 (51.0)	47.3 (41.8–52.8)	2.8	43.1	27.5 (18.6–38.5)	65.2 (54.1–74.9)	84.0 (74.2–90.5)

Notes: S.E. = Standard error.; * *N* = unweighted number of subjects for the whole year.

**Table 3:** Seasonal population-weighted (age, sex) serum 25(OH)D levels and the prevalence of serum 25(OH)D levels <30, <50, and <75 nmol/L (95% CI) for the adult (18–64 years) and elderly (65–74 years) populations.

	Extended Summer: May–October							Extended Winter: November–April						
	*N* (%) *	Serum 25(OH)D Level (nmol/L)			Prevalence (%)			*N* (%) *	Serum 25(OH)D Level (nmol/L)			Prevalence (%)		
		Mean	S.E.	Med.	<30 nmol/L	<50 nmol/L	<75 nmol/L		Mean	S.E.	Med.	<30 nmol/L	<50 nmol/L	<75 nmol/L
Adults	52 (100)	70.4 (62.2–78.5)	4.1	64.0	2.6 (0.6–10.2)	25.3 (14.8–39.9)	62.6 (47.4–75.6)	73 (100)	36.7 (32.5–40.9)	2.1	34.4	40.8 (29.0–53.7)	81.6 (69.4–89.7)	98.0 (92.2–99.5)
- Male	22 (42.3)	76.2 (62.4–90.1)	6.9	71.2	2.8 (0.3–18.3)	16.1 (4.9–41.5)	56.6 (34.1–76.6)	30 (41.1)	40.3 (33.3–47.3)	3.5	37.6	37.1 (20.4–57.6)	74.2 (53.3–87.8)	96.0 (84.9–99.0)
- Female	30 (57.7)	64.5 (56.5–72.5)	4.0	63.0	2.4 (0.3–15.8)	34.6 (19.5–53.6)	68.5 (48.8–83.3)	43 (58.9)	33.1 (28.8–37.4)	2.2	32.8	44.5 (29.7–60.3)	89.1 (76.9–95.3)	100
Elderly	64 (100)	60.1 (54.0–66.2)	3.1	57.0	7.8 (3.2–17.5)	40.2 (28.8–52.7)	73.4 (61.1–82.9)	91 (100)	39.0 (35.0–43.0)	2.0	37.4	34.6 (25.4–45.1)	78.8 (69.0–86.1)	92.2 (84.4–96.3)
- Male	29 (45.3)	62.6 (54.4–70.8)	4.1	58.5	6.9 (1.7–24.1)	27.6 (14.3–46.5)	72.4 (53.5–85.7)	47 (51.7)	38.2 (33.3–43.0)	2.4	38.1	27.7 (16.7–42.2)	83.0 (69.3–91.3)	93.6 (81.8–98.0)
- Female	35 (54.7)	57.9 (49.0–66.9)	4.5	47.9	8.6 (2.7–23.8)	51.4 (35.1–67.5)	74.3 (57.2–86.2)	44 (48.3)	39.8 (33.6–46.0)	3.1	36.5	40.9 (27.4–56.0)	75.0 (60.1–85.7)	90.9 (78.0–96.6)

Notes: med. = median; * *N* = unweighted number of subjects per season.

**Table 4:** Sample prevalence of serum 25(OH)D levels below 50 nmol/L, and adjusted odds ratios (95% CI) for adult (18–64 years) and elderly (65–74 years) population.

Variable		Adults			Elderly		
		*N*	Prevalence *N* (%)	Odds Ratio	*N*	Prevalence *N* (%)	Odds Ratio
Overall		125	73 (58.4)		155	98 (63.2)	
Place of living	village	63	35 (55.6)	1.80 (0.57–5.64)	84	52 (61.9)	0.49 (0.18–1.35)
	town	19	14 (73.7)	3.77 (0.58–24.51)	24	15 (62.5)	0.87 (0.24–3.15)
	city	43	24 (55.8)	1	47	31 (66.0)	1
Sex	male	52	25 (48.1)	1	76	47 (61.8)	1
	female	73	48 (65.8)	3.36 (1.05–10.74)	79	51 (64.56)	1.94 (0.80–4.69)
Education	elementary school	11	7 (63.6)	3.23 (0.47–22.19)	30	23 (76.7)	1.16 (0.33–4.00)
	high school	75	42 (56)	1	86	53 (61.6)	1
	higher education	39	24 (61.5)	2.19 (0.58–8.23)	39	22 (56.4)	0.56 (0.21–1.54)
Family net income *	≤900 €	23	14 (60.9)	1.57 (0.34–7.34)	48	34 (70.8)	1.75 (0.65–4.76)
	900–1800 €	54	31 (57.4)	1	82	49 (59.8)	1
	>1800 €	36	21 (58.3)	1.78 (0.51–6.25)	19	13 (68.4)	1.47 (0.38–5.65)
Season	November–April	73	59 (80.8)	34.94 (9.22–132.50)	91	72 (79.1)	10.34 (4.10–26.07)
	May–October	52	14 (26.9)	1	64	26 (40.6)	1
BMI	<25	49	31 (63.3)	1.68 (0.52–5.35)	46	24 (52.2)	0.31 (0.12–0.78)
	≥25	76	22 (51.2)	1	109	34 (56.7)	1
Smoking status	current smoker	22	15 (56.3)	1	18	11 (63.5)	1
	ex-/non-smoker	103	58 (68.2)	5.01 (0.90–27.78)	137	87 (61.1)	1.40 (0.36–5.43)
Physical activity	low level	39	28 (71.8)	5.59 (1.38–22.69)	51	33 (64.7)	1.78 (0.66–4.81)
	moderate level	40	20 (50.0)	1	49	28 (57.1)	1
	high level	46	25 (54.4)	1.76 (0.44–7.05)	54	36 (66.7)	1.57 (0.59–4.22)
Vitamin D supplement use	users	11	5 (45.5)	1	13	7 (53.9)	1
	non-users	114	68 (59.7)	1.20 (0.17–8.25)	142	91 (64.1)	3.61 (0.82–15.92)

Notes: * Logistic regression analysis conducted on samples with the excluded missing data (family net income: *n* = 12 (adults) and *n* = 6 (elderly)); variables contributing significantly to the variability in the distribution between the different 25(OH)D serum levels (adults: sex (*p* = 0.041), season (*p* < 0.0001), physical activity (*p* = 0.048; low level of physical activity: *p* = 0.016); elderly: season (*p* < 0.0001); BMI (*p* = 0.014).

## Supplementary Materials

The following are available online at https://www.mdpi.com/2072-6643/12/6/1838/s1. Table S1: Sample mean (95% CI) serum 25(OH)D levels (nmol/L) for the different groups of the adult and elderly population.
