# Supplementary material for: Nutrihealth Study: Seasonal Variation in Vitamin D Status Among the Slovenian Adult and Elderly Population
Source: Nutrients. 2020 Jun 19;12(6):1838. doi: 10.3390/nu12061838 (PMC7353282; doi:10.3390/nu12061838)
Supplement: Supplementary file 1 [file nutrients-12-01838-s001.pdf]

Hribar et al. 2020. **Nutrihealth Study: Seasonal Variation in Vitamin D Status Among the Slovenian Adult and Elderly Population**

Supplementary Materials Table S1. Sample mean (95% CI) serum 25(OH)D levels (nmol/L) of adults and the elderly according to different variables.

Variable		Adults (18–64 years)			Elderly (65–74 years)		
		N	Unadjusted	Adjusted	*N*	Unadjusted	Adjusted
Overall		125	49.9 (45.3–54.6)		155	47.7 (43.9–51.4)	
Residential area	village	63	51.2 (43.8–58.7)	47.8 (42.6–53.0)	84	47.4 (41.8–53.0)	49.1 (44.4–53.8)
	town	19	46.1 (36.8–55.3)	51.4 (42.0–60.9)	24	52.4 (42.4–62.5)	48.7 (40.1–57.4)
	city	43	49.8 (42.3–57.3)	51.2 (45.1–57.2)	47	45.6 (39.7–51.5)	42.9 (36.5–49.3)
Sex	male	52	55.5 (47.2–63.8)	55.0 (48.9–60.8)	76	47.5 (42.4–52.6)	48.6 (43.8–53.5)
	female	73	46.0 (40.6–51.3)	46.2 (41.6–50.9)	79	47.8 (42.2–53.5)	45.7 (40.9–50.5)
Education	primary school	11	41.8 (27.3–56.4)	38.5 (25.8–51.1)	30	38.4 (29.6–47.2)	42.6 (34.4–50.8)
	high school	75	51.0 (40.6–59.7)	50.7 (46.0–55.4)	86	49.9 (44.7–55.1)	47.4 (42.8–52.0)
	higher education	39	50.2 (40.6–59.7)	50.7 (43.9–57.5)	39	49.8 (43.0–56.7)	50.2 (49.9–57.4)
Family net income*	≤900 €	23	45.6 (38.0–53.1)	49.0 (40.3–57.7)	48	43.2 (36.0–50.3)	44.6 (38.3–50.9)
	900–1800 €	54	50.8 (42.8–58.9)	51.0 (45.7–56.3)	82	50.0 (44.9–55.1)	49.2 (44.7–53.8)
	>1800 €	36	50.3 (42.5–58.2)	47.8 (41.3–54.4)	19	43.6 (34.1–53.0)	44.7 (34.4–55.1)
Season	November-April	73	36.7 (32.8–40.7)	36.0 (31.4–40.6)	91	39.0 (35.0–42.9)	38.0 (33.5–42.4)
	May-October	52	68.5 (61.2–78.8)	70.2 (64.4–75.9)	64	60.0 (53.9–66.2)	60.3 (54.9–65.6)
BMI	<25	49	50.8 (43.5–58.0)	51.6 (45.4–57.9)	46	54.0 (45.7–62.4)	54.9 (48.5–61.4)
	≥25	76	49.4 (43.2–55.6)	48.3 (43.6–53.1)	109	45.0 (40.9–49.0)	44.0 (40.0–48.0)
Smoking status	current smoker	22	44.0 (33.1–54.8)	38.4 (29.1–47.7)	18	46.7 (36.1–57.2)	43.8 (33.4–54.1)
	ex-/non-smoker	103	51.2 (46.0–56.4)	52.0 (48.0–56.0)	137	47.8 (43.7–51.9)	47.6 (44.1–51.1)
Physical activity	low level	39	43.9 (36.5–51.4)	45.6 (39.3–51.9)	51	45.7 (39.2–52.3)	44.3 (38.5–50.2)
	moderate level	40	54.1 (45.8–62.4)	54.6 (47.9–61.3)	49	50.7 (43.2–58.2)	49.8 (43.9–55.7)
	high level	46	51.4(42.8–60.0)	42.5 (40.9–55.3)	54	47.2 (41.2–53.3)	47.3 (41.7–53.0)
Vitamin D supplement use	users	11	55.6 (34.6–76.6)	49.9 (36.2–63.6)	13	54.0 (40.6–67.5)	57.3 (45.3–69.2)
	non-users	114	49.4 (44.6–54.2)	49.5 (45.9–53.3)	142	47.0 (43.1–51.0)	46.8 (42.7–49.7)

Notes: * Linear regression analysis conducted on samples with excluded missing data (Family net income: n = 12 (adults) and n = 6 (elderly)); identified predictors accounting for variability in the serum 25(OH)D concentration (adults: sex ($p = 0.031$), season ($p < 0.001$), and smoking status ($p = 0.011$); Elderly: season ($p = <0.001$), BMI ($p = 0.0065$)
